# Assessing the applicability of cerebrospinal fluid collected from the spinal cord for the determination of ethyl alcohol in post-mortem toxicology

**DOI:** 10.1007/s12024-022-00560-8

**Published:** 2022-11-28

**Authors:** Paulina Wachholz, Rafał Skowronek, Natalia Pawlas

**Affiliations:** 1 Department of Pharmacology, Medical University of Silesia in Katowice, Faculty of Medical Sciences in Zabrze, 38 Jordana Street, 41-808 Zabrze, Poland; 2 Toxicology Laboratory ToxLab, 6 Kossutha Street, 40-844 Katowice, Poland; 3 Department of Forensic Medicine and Forensic Toxicology, Faculty of Medical Sciences in Katowice, Medical University of Silesia in Katowice, 18 Medyków Street, 40-752 Katowice, Poland

**Keywords:** Cerebrospinal fluid, Ethyl alcohol, Forensic toxicology, Alternative biological specimens

## Abstract

This paper presents the results of a study on the applicability of cerebrospinal fluid (CSF) collected from the spinal canal in the post-mortem determination of ethyl alcohol. The present study reviewed data of autopsy cases (*n* = 45), in which ethyl alcohol was detected in CSF using gas chromatography with a flame ionization detector (HS-GC-FID), to investigate ethyl alcohol concentrations in CSF, compared with blood. As a result of statistical analysis of the obtained data, a high positive correlation was found between blood ethanol concentration and cerebrospinal fluid collected from the spinal canal ethanol concentration. The Pearson correlation coefficient was statistically highly significant (*p* < 0.001) (*r* = 0.9503). The data obtained allowed us to conclude that cerebrospinal fluid collected from the spinal canal can be collected during an autopsy as an alternative biological specimen to assess the ethanol content. Cerebrospinal fluid collected from the spinal canal can corroborate and lend credibility to the results obtained for blood and, in special cases, when blood is drawn from putrefied bodies and may even be a superior specimen to blood for assessing ethyl alcohol intoxication status.

## Introduction

Post-mortem toxicological testing for ethanol in body fluids is one of the most important components accompanying forensic autopsies around the world. Such examinations are routinely performed, and their results are often conclusive in determining the circumstances and cause of death [1]. One of the key steps that can pose difficulties during diagnosis and interpretation of the ethyl alcohol-intoxicated state at the time of death is securing biological specimens for toxicological testing [2]. For example, in Poland, this should be done according to the Recommendations of the Polish Society of Forensic Medicine and Criminology on the collection of autopsy specimens for toxicological testing [3]. These guidelines date back to 2012 and have not been updated since, despite suggestions that they are now outdated and need to be revised [4]. It is also worth mentioning that there is a large amount of ongoing research into the potential use of alternative biological specimens in toxicological analysis, including although not limited to the use of costal cartilage in the post-mortem diagnosis of ethyl alcohol intoxication [5]. The advantages, disadvantages, and possibility of using CSF as an alternative biological specimen have been recently reviewed [6].

The primary biological material that should be secured at autopsy is peripheral blood, which is drawn directly from the femoral vein. Unfortunately, under certain circumstances, it is not possible to collect such blood samples, for example, when there has been an extensive injury and/or exsanguination [7]. An example of an extensive injury, where there is an insufficient amount of autopsy blood for testing, is cases of marked head injuries. This is a result of peripheral blood shunting rather than exsanguination [8]. It should also be taken into account that blood is susceptible to biochemical and post-mortem changes, and interpreting the result in a putrefied body may need additional testing by simultaneous examination of other body fluids. This procedure aims to exclude the potential presence of endogenous ethanol in the body, which may reach values as high as, and sometimes exceeding, 0.5 g/L of alcohol in the blood, thus carrying the risk of an incorrect assessment [9, 10]. Therefore, in addition to peripheral blood, simultaneous collection of vitreous humor and/or urine is useful [9, 10]. There has not yet been any extensive research on the routine use of cerebrospinal fluid collected directly from the spinal cord for this purpose. Data on the use of cerebrospinal fluid collected from different sites for forensic toxicology testing of ethanol content are inadequate as they refer to the determination of ethanol by methods that are not currently used and were subject to a high error risk, such as the classic Widmark method which can give false-positive results in, for example, diabetic patients. We do not dismiss entirely the early studies on ethanol in CFS, but there is some evidence that the classical Widmark method is not specific for ethanol, because some other components (for example, amines, amides, ether, acetone, ketone bodies) can reduce bichromate [11]. The previous studies that were conducted with gas chromatography (GC) solely involved analysis of cerebrospinal fluid derived from the brain ventricles [12–14]. The above observations are significant since cerebrospinal fluid collected from the ventricular system of the brain differs in biochemical composition from that collected from the spinal cord. Both of these anatomical locations cannot be treated equally until more data is collected [15]. Fluid from the ventricular system has one significant disadvantage—it is not sufficiently isolated and therefore undergoes greater post-mortem changes than fluid collected from the spinal cord. Cerebrospinal fluid in the subarachnoid space around the spinal column may be more stagnant owing to gravitational effects, whereas in the ventricles, CSF may have a higher cellular turnover. The brain is a highly cellular and metabolically active organ [15]. Brain tissue decomposes and putrefies right after death. Sometimes, a collection of the clean CSF from the ventricles was not possible because of the highly softened or liquefied consistency of the brain tissues, so it does not necessarily refer to the decay process and the formation of endogenous alcohol in this fluid, but to its contamination and the quality of the sample. Due to the proximity of the brain, the cerebrospinal fluid from the ventricular system may be contaminated more quickly than the one from the spinal cord, even from the top of the spinal column, because fluid from the spinal cord is cleaned as more samples are taken. Collecting CSF from increasingly deeper layers of the spinal canal makes the contaminated sample purer, as deeper layers of fluid are not contaminated due to lack of movement. What is more, when one sometimes tries to collect CSF, it appears to be contaminated with blood. The reason for this contamination may be subarachnoid bleeding or intracerebral hemorrhage [6]. Certain sites in the body (vitreous, CSF) are more sequestered (or have less glucose) than the vasculature, and accordingly, it is more difficult (or takes longer) for bacteria to get into these areas and then produce ethanol. The authors’ observations show that due to the injuries, the fluid from the ventricles is more often contaminated with blood than fluid from the spinal canal.

These reports provided the inspiration for observations and preliminary studies of cerebrospinal fluid collected directly from the spinal canal specifically to assess its usefulness in the post-mortem diagnosis of ethyl alcohol intoxication.

## Materials and methods

### Collection of biological specimens

The specimens for the study consisted of blood and cerebrospinal fluid samples collected during forensic autopsies in 2019–2022 conducted in Silesia and Masovia in Poland, following their having obtained a positive opinion from the Bioethics Committee of the Silesian Medical University in Katowice (decision no. PCN/0022/KB/2/19). Statistical analysis was conducted on cases whereby ethanol was found in the blood, CSF, and biological specimens which did not yet show signs of putrefaction that were collected at autopsies (*n* = 45). An additional group of samples consisted of cases in which the presence of ethanol was found only in the blood (*n* = 8), plus the specimens were collected during autopsy in which putrefactive processes had already begun.

Blood samples were collected directly from a femoral vein, whereas cerebrospinal fluid samples were collected from the spinal cord by controlled aspiration with a 12-cm-long needle after opening the cranial cavity and moving aside the encephalon. This procedure appears to be one of the simplest ones, although it bears the risk of contaminating the sample with blood. The blood and cerebrospinal fluid samples obtained were 5 mL in volume and were collected into test tubes containing the preservative (1–2% sodium fluoride (NaF)). Until analysis, the samples were stored in a refrigerator at + 4 °C (the monitoring of the temperature in the refrigerators was conducted by daily readings).

### Preparation of samples for testing

After removal from the refrigerator, the blood and cerebrospinal fluid tubes were rotated several times to make them homogeneous before collection. Sample preparation for ethanol testing included collecting 100 µl of the test sample and 400 µl of the internal standard (*t*-butanol 1.5 g/L), as is recommended [16], before being then added to 20-mL disposable glass vials. Once the sample and internal standard were added to the vials, they were immediately crimped with aluminum caps with rubber septa.

### Chromatographic analysis

Each sample was analyzed twice via headspace gas chromatography with flame ionization detection (HS-GC-FID) on two columns of different polarity (Column A: Restek Rtx-BAC PLUS 1 0.32 mm × 30 m × 1.8 µm; Column B: Restek Rtx-BAC PLUS 2 0.32 mm × 30 m × 0.6 µm), while ensuring different retention times for ethanol and the internal standard on each column, in order to exclude the possibility of biasing the results by the presence of possible post-mortem volatile compounds. The samples were analyzed using a Thermo Scientific TRACE GC with a TriPlus 300 autosampler connected by a transfer line under isothermal conditions. The oven and transfer line temperatures in the headspace autosampler were 85 ℃ and 100 ℃, respectively. The injector temperature was 200 °C. The split ratio was 10:1. The column temperature was 40 °C. The carrier gas was helium (12 mL/min). The software employed was Chrom-Card. All of the measurements were performed in a certified laboratory, Toxlab (Certificate of Proficiency issued by DAkkS no. Et 2/20).

In Polish law, the necessary conditions for the method to determine evidence-useful blood alcohol concentrations is to maintain the total error below ± 0.05 g/L for concentrations up to 1.0 g/L and 5% for concentrations above 1.0 g/L. Our method meets these requirements.

To assess method linearity, a seven-level calibration curve (with concentrations ranging from 0.10 to 5.0 g/L) was used with the internal standard (t-butanol) to ensure the accuracy of the responses. Linearity and peak area repeatability as indicated by the low level RSD% show the capability of this system to provide quality results for this analysis (see Table 1).

**Table 1:** Validation parameters

Parameter	Value	
	Column A	Column B
Coefficient of determinations R2	0.99993	0.99987
The peak area repeatability (as % RSD) of the internal standard (1.5 g/L t-Butanol) for *n* = 10 injections	1.53	1.77
The peak area repeatability (as % RSD) of the ethanol (1 g/L) for *n* = 10 injections	0.98	1.05

### Statistical analysis

For the statistical analysis, the average of the ethanol concentrations obtained for a given sample from two injections on each of the two columns of different polarity was used. The normality of the distribution of ethanol concentrations in the analyzed body fluids was tested by the Shapiro–Wilk test. The correlation between these body fluids was further investigated by the correlation and linear regression method, assuming a significance level of *p* < 0.05.

## Results

**Fig. 1:**
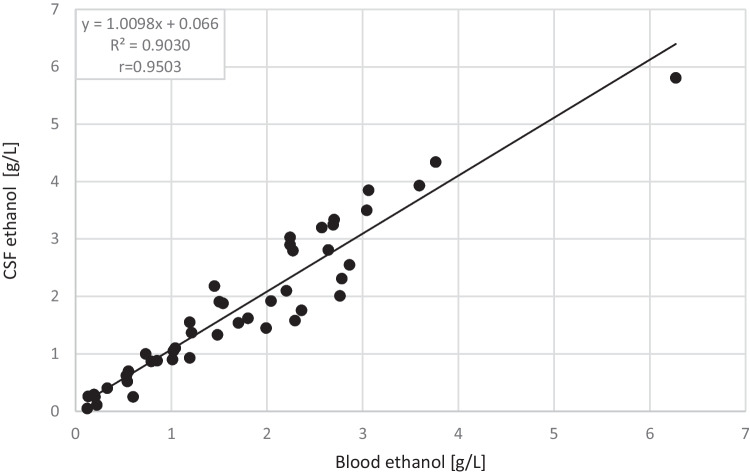
Correlation of ethyl alcohol concentrations in blood and cerebrospinal fluid

While comparing the results of ethyl alcohol concentrations in blood with the results of ethyl alcohol concentrations in cerebrospinal fluid, a strong positive correlation was found between them (*r* = 0.9503, *p* < 0.001) (see Table 2). The following table shows the results of the mean and median for the concentrations of ethanol in blood and cerebrospinal fluid, their differences, and their ratios. The resulting equation for the linear regression model took the form of *ny* = 1.0098*x* + 0.0066, where *y* is the concentration of ethanol in CSF, and *x* is the concentration of ethanol in the blood (see Fig. 1).

**Table 2:** Test results for the entire group of cases studied and the value of the correlation coefficient

	Blood (B)	Cerebrospinal fluid (CSF)	Difference between blood and CSF alcohol concentrations	Ratio of B:CSF concentrations
Number of cases (n) = 45				
Range of concentration variation (g/L)	0.12 to 6.27	0.05 to 5.81	− 0.79 to 0.75	0.5 to 2.4
Mean (*x*)	1.74	1.82	− 0.08	1.04
Median (Me)	1.54	1.58	− 0.04	1.02
Residual standard deviation (SD)	± 0.408			
Standard error (SE)	± 0.0608

**Fig. 2:**
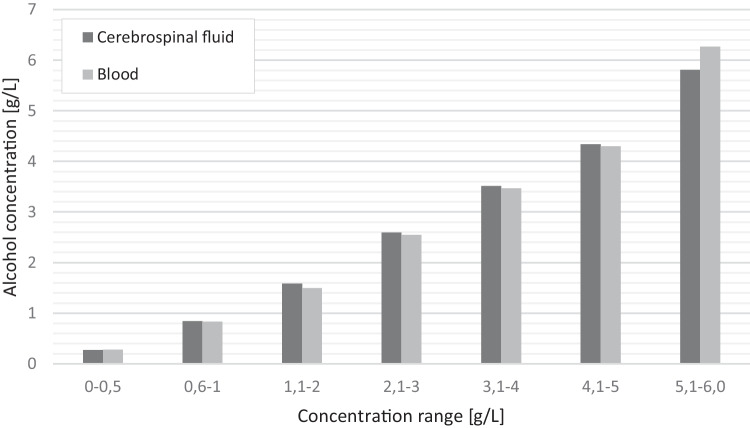
Comparison of average ethanol concentrations in blood and cerebrospinal fluid including the concentration range

The majority (*n* = 27; 60%) of cases showed higher alcohol concentration in CSF than in blood. In 14 cases (31%), the alcohol concentration in CSF was lower than in blood. In the other cases (*n* = 4; 9%), the alcohol concentration in CSF and blood were qual. As shown in Fig. 2, only in the ranges of 0.1–0.5 g/L and 5.1–6.0 g/L, the average alcohol concentration in CSF was lower than in blood.

An additional group of samples, not included in the statistical analysis, were cases in which ethanol was determined only in the blood (*n* = 8). A positive result was considered to be an alcohol concentration greater than or equal to LOD (0.05 g/L). In this group of samples, putrefactive processes had already begun, but the presence of n-propanol was detected in one case only. There was no evidence of ethanol in the cerebrospinal fluid and in the vitreous in this group of samples (see Table 3).

**Table 3:** Examples of results of alcohol content in blood in cases which ethanol was determined neither in cerebrospinal fluid nor in vitreous

Case No.	The alcohol concentration in blood (g/L)	State of decomposition/historical information
1	0.05	Slightly/COVID-19
2	0.05	Slightly/myocardial infarction
3	0.07	Slightly/natural death
4	0.11	Moderately/without history
5	0.13	Moderately/without history
6	0.20	Totally/without history
7	0.21	Slightly/blunt trauma
8	0.47 (additionally n-propanol 0.11 g/L)	Moderately/suicide by hanging

## Discussion

The found linear regression model should not be used to predict ethanol content in blood from the determined ethanol content in CSF or conversely, as this would involve a magnitude of uncertainty in the result obtained. The greatest difference between the obtained ethanol results in the blood-cerebrospinal fluid sample pair was as high as 0.79 g/L, whereas the lowest was only 0.03 g/L. Thus, it is important to understand that, depending on the pharmacokinetic phase that occurred at the time of death, these differences may be significant and cannot always be clearly described by a simple linear regression model. Possible explanations of this phenomenon are the widespread assumption that alcohol diffuses according to Fick’s law by simple diffusion and that alcohol, during the absorption phase, diffuses through body fluids according to the blood-cerebrospinal fluid-vitreous humor-perilymph pattern, while during the elimination phase, the order is reversed [17]. The results of the above-mentioned study confirmed the previous report on the examination of cerebrospinal fluid, which was collected from the brain ventricles. The authors have also indicated a very high correlation between concentrations of alcohol in the blood and the cerebrospinal fluid [13]. This implies that, despite differences in the biochemical composition of cerebrospinal fluid collected from the spinal cord and those collected from the brain ventricles, their usefulness in post-mortem diagnosis of ethyl alcohol intoxication is comparable, and regardless of the site of collection, cerebrospinal fluid can be a valuable alternative biological specimen. The collection of cerebrospinal fluid from the spinal cord can be particularly useful in cases where post-mortem processes resulting in the synthesis of endogenous chemical compounds have already begun in the blood that can falsify the result of a test for ethanol or where it is not possible to collect blood due to, for instance, exsanguinations. The determination of the pharmacokinetic phase in such cases is of secondary importance, and what matters primarily is whether ethanol was present in the body at all at the time of death and what the order of magnitude of the ethanol concentration in the body was at that time.

Regarding the group of samples where alcohol was determined only in the blood, in the first seven cases, the concentration of alcohol in the blood was not significant, and in the situation of obtaining information about the initiated putrefaction, the interpretation of such cases does not pose any difficulty. However, the final case may provide evidence that cerebrospinal fluid, when compared to blood, is much less susceptible to biochemical and post-mortem changes, until it becomes contaminated with blood due to the breakdown of the membrane surrounding the spine. Furthermore, the n-propanol (0.11 g/L) was also determined in the last blood sample. This suggests post-mortem microbial production of ethanol [18]. The possibility of producing endogenous ethanol in the body after death has always been and still is an issue when concentrations of ethanol in post-mortem specimens are interpreted [19]. Ethyl alcohol could be formed post-mortem in various and non-predictable amounts, at a different time from death and different extents of putrefaction, as a function of the numerous variables such as temperature, type, and number of microorganisms present either in corpses or specimens collected at autopsy or the cause of death. Through the accurate study of these cases and additional analysis of vitreous and cerebrospinal fluid, we were able to conclude that alcohol found in these 8 cases was considered to be of endogenous production. Vitreous is strongly recommended as a body fluid for the determination of ethanol in post-mortem toxicology, but less common autopsy specimens like cerebrospinal fluids can also be helpful, as we confirmed in these cases.

## Conclusions

The data obtained preliminarily suggests that cerebrospinal fluid, regardless of the collection site, can be used to determine the ethanol content at the time of death. Cerebrospinal fluid may be routinely collected during forensic autopsies, particularly when other body fluids cannot be collected, or putrefactive processes have already begun in the body.

Analysis of cases in which samples of biological specimens showed signs of putrefaction processes allows us to conclude that cerebrospinal fluid collected from the spinal canal is less likely to undergo post-mortem transformations, and its analysis may be helpful in cases of disputable issues concerning the state of ethyl alcohol intoxication at the time of death, until it becomes contaminated with blood due to the breakdown of the membrane surrounding the spine.

## Key points

1. Cerebrospinal fluid can be used to determine ethanol content at the time of death.
2. Cerebrospinal fluid may be routinely collected during forensic autopsies, particularly when other body fluids are unavailable.
3. Cerebrospinal fluid collected from the spinal canal is less likely to undergo post-mortem transformations (putrefactive processes), until it becomes contaminated with blood due to the breakdown of the membrane surrounding the spine.
